# Death-Associated Protein Kinase 1 Inhibits Progression of Thyroid Cancer by Regulating Stem Cell Markers

**DOI:** 10.3390/cells10112994

**Published:** 2021-11-03

**Authors:** Mi-Hyeon You, Woo Kyung Lee, Meihua Jin, Dong Eun Song, Sheue-yann Cheng, Tae Yong Kim, Won Bae Kim, Min Ji Jeon, Won Gu Kim

**Affiliations:** 1 Department of Internal Medicine, Asan Medical Center, University of Ulsan College of Medicine, Seoul 05505, Korea; mhyou80@gmail.com (M.-H.Y.); meihua711@naver.com (M.J.); tykim@amc.seoul.kr (T.Y.K.); kimwb@amc.seoul.kr (W.B.K.); 2 Laboratory of Molecular Biology, Center for Cancer Research, National Cancer Institute, National Institutes of Health, Bethesda, MD 20892, USA; wookyung.lee@nih.gov (W.K.L.); Chengs@nih.gov (S.-y.C.); 3 Department of Pathology, Asan Medical Center, University of Ulsan College of Medicine, Seoul 05505, Korea; hipuha@hanmail.net

**Keywords:** *DAPK1*, thyroid cancer, neoplastic stem cells, tumor suppressor

## Abstract

The activation of metastatic reprogramming is vital for cancer metastasis, but little is known about its mechanism. This study investigated the potential role of death-associated protein kinase 1 (DAPK1) in thyroid cancer progression. We generated knockdown (KD) DAPK1 using siRNA or shRNA in 8505C and KTC-1 cell lines, which we transiently or stably overexpressed in MDA-T32 and BCPAP cell lines. DAPK1 KD in 8505C and KTC-1 cells significantly increased cell proliferation and colony formation compared with controls. We observed significant inhibition of cancer cell invasion in cells overexpressing DAPK1, but the opposite effect in KD cells. Tumorsphere formation significantly increased after inhibition of DAPK1 expression in 8505C cells and was significantly suppressed in DAPK1-overexpressing MDA-T32 and BCPAP cells. DAPK1 overexpression inhibited mRNA and protein levels of stem markers (OCT4, Sox2, KLF4, and Nanog). Furthermore, the expression of these markers increased after KD of DAPK1 in 8505C cells. Mechanistic studies suggest that DAPK1 may modulate the expression of stem cell markers through the inhibition of β-catenin pathways. These findings were consistent with the public data and our thyroid tissue analysis, which showed higher DAPK1 expression was associated with advanced-stage papillary thyroid cancer with a higher stemness index and lower disease-free survival.

## 1. Introduction

Metastasis is a multi-step process that causes cancer cells to migrate from their primary site and progress into secondary tumors in other organs [1]. Cancer stem cells (CSCs) are a small subset of tumor cells with self-renewal and differentiation characteristics. CSCs are important during cancer invasion and metastasis with roles implicated in metastasis-initiation through their genetic signatures, and in angiogenesis and lymphangiogenesis, which are essential for metastasis [2]. Some CSCs also have epithelial-to-mesenchymal transition (EMT)-like features that are important [3]. The association between CSCs and EMTs is a key variable in drug resistance and poor clinical outcomes in cancer [4,5,6].

DAPK1 is a death-domain-containing calcium/calmodulin-regulated serine/threonine kinase through functional replication in interferon-γ-induced apoptosis [7]. DAPK1 plays an important role in apoptosis, autophagy, the immune response, ischemic injury of the brain, and neurodegenerative disease [8,9]. It has also been identified as a tumor suppressor, and several studies have shown that DAPK1 regulates cell adhesion, DNA repair, cell cycle checkpoint control, and nuclear receptors during cancer progression [10,11,12,13,14]. Recently, reports have emerged suggesting that DAPK1 may closely interact with CSCs in colon, breast, and gastric cancer [12,13,15]. One study reported that downregulation of DAPK1 promoted stem cell expression through modulating Ca^2+^ channel activation [13]. Other studies have demonstrated that downregulation of DAPK1 promotes CSCs by activating ZEB1 in colon and prostate cell lines [16,17]. Additionally, hypermethylation of DAPK1 has been a prognostic indicator in patients with various cancers [12,14,18,19,20]. However, its role in thyroid cancer has not yet been determined.

In this study, we evaluated the role of DAPK1 in thyroid cancer progression. First, we assessed DAPK1 mRNA expression in The Cancer Genome Atlas (TCGA). We observed a significant decrease of DAPK1 in advanced-stage papillary thyroid cancer (PTC) tissues and associated disease-free survival (DFS). Therefore, we evaluated the potential role of DAPK1 as a tumor suppressor in thyroid cancer.

## 2. Materials and Methods

### 2.1. Analysis of TCGA Data

A transcriptome and its matched clinicopathological data of TCGA-thyroid cancer (THCA) were obtained from Broad GDAC Firehose (https://gdac.broadinstitute.org/, accessed on 5 January 2020) and cBioPortal for Cancer Genomics (https://www.cbioportal.org/, accessed on 5 January 2020), and we then explored the biological and clinical significance of the DAPK1 gene in the dataset. The dataset was divided into two groups according to high (upper half) or low (lower half) DAPK1 mRNA expression. The machine-learning-generated SI [21,22], and clinicopathological indicators such as tumor size, TNM stage, and DFS were then compared between these two groups. For these clinical analyses, we used IBM SPSS Statistics 24 (IBM Inc., Armonk, NY, USA) and GraphPad Prism 8 (GraphPad Software Inc., San Diego, CA, USA).

### 2.2. Cell Culture

The BCPAP, MDA-T32, and KTC-1 cells originated from PTC with a BRAF mutation, while the origin of the 8505C was anaplastic thyroid cancer (ATC) with BRAF mutation. TPC-1 cells originated from PTC with RET/PTC1 rearrangement, and BCPAP, MDA T32, 8505C, KTC-1, and TPC-1 cells were maintained in RPMI 1640 medium (GIBCO, Grand Island, NY, USA) containing 10% fetal bovine serum (FBS), (GIBCO, Grand Island, NY, USA) [23]. The 293FT cells were purchased from the American Type Culture Collection and maintained in low-glucose Dulbecco’s modified Eagle’s medium supplemented with 10% FBS (Life Technologies, Grand Island, NY, USA). Cultures were maintained at 37 °C under 5% CO_2_.

### 2.3. Plasmid Transfection

We used the construct encoding human the DAPK1 Vector and DAPK1 ΔCaM mutant (with a deleted Ca^2+^/calmodulin regulatory domain) with a myc-tag in these experiments [8]. According to the manufacturer’s instructions, cells were transiently transfected with different plasmids using Lipofectamine 3000 transfection reagent (Invitrogen, Carlsbad, CA, USA). We generated stable DAPK1-overexpressing MDA-T32 and BCPAP cells by transfecting a GFP-DAPK1 clone constructed in a lentiviral Plko-puro vector into 293FT cells with MD2 and pax2 expression plasmids. Sixty hours after transfection, the virus-containing supernatant was used to infect MDA-T32 and BCPAP cells. Stable clones were selected using 2–4 μg/mL puromycin 1 day after infection. We generated shRNA-DAPK1 in 8505C and KTC-1 cells by performing a Flag shRNA transfection with different sequences. The target sequences were CCGGCCACGTCGATACCTTGAAATTCTCGAGAATTTCAAGGTATCGACGTGGTTTTT (TRCN0000000983, shDAPK1#1) and CCGGCCACGTCGATACCTTGAAATTCTCGAGAATTTCAAGGTATCGACGTGGTTTTTG (TRCN0000284935, shDAPK1#2) for the human DAPK1 gene.

### 2.4. Small Interfering RNA Transfection

Four small interfering RNAs (siRNAs) to human DAPK1 or scrambled siRNAs were transfected with 8505C and KTC-1 using Lipofectamine 3000 Reagent (Invitrogen, Carlsbad, CA, USA) according to the manufacturer’s instructions. Briefly, the control and experimental groups were seeded into 6-well plates at 70–80% confluence. Cells in each well were then transfected with 10 μL of 20 μM siRNA. After 12–24 h of transfection, the medium was replaced with a new medium and used for various experiments.

Si-RNAs specific for DAPK1 and scrambled si-RNAs were purchased from DHARMACON (Lafayette, CO, USA) and are described as follows:

Human DAPK1-#1, GAAUGGAGUUGGCGAUUUC;
Human DAPK1-#2, GUUUGUCGCUCCUGAGAUA;
Human DAPK1-#3, AUACGAAGCCAGAUUGUUU;
Human DAPK1-#4, AUACUACAGUUGCUCAUUA.
Those for the scrambled si-RNAs were as follows:
Human Control-#1, UAGCGACUAAACACAUCA;
Human Control-#2, UAAGGCUAUGAAGAGAUAC;
Human Control-#3, AUGUAUUGGCCUGUAUUAG;
Human Control-#4, AUGAACGUGAAUUGCUCAA.

The scrambled si-RNAs did not match any human sequences in a Gene Bank search.

### 2.5. Cell Proliferation Assay

The effect of DAPK1 KD or overexpression on cell proliferation in 8505C, KTC-1, MDA-T32, and BCPAP cells was measured by the 3-(4, 5-dimethylthiazol-2-yl)-2, 5-diphenyltetrazolium bromide assay (MTT assay; Cell Bio labs, San Diego, CA, USA). Briefly, cells were treated in 96-well plates for different periods (1, 3, and 5 days). MTT reagents were added to each well, followed by DMSO after 4 h to dissolve MTT in the cells. Dissolved MTT was then measured at 595 nm using the ELISA method.

### 2.6. Colony-Forming Assay

Each cell line (BCPAP, MDA-T32, 8505C, KTC-1, and TPC-1) was divided into 6-well plates at a density of 5 × 103 cells per well in 2 mL of medium for each experiment with control cells. Cell medium was changed after 24 h, and every 3 days thereafter. On day 7, media was stained with 0.1% crystal violet in 20% methanol. After acquiring the image, the statistical significance of the cell proliferation was determined by measuring absorbance at 595 nm after dissolving the media in 1 mL of DMSO.

### 2.7. Matrigel Invasion Assay

Matrigel Invasion Chambers (BD Bioscience, Bath, UK) were used to assess the invasion capacity of each experimental and control group as previously described [23]. Briefly, matrigel coating was performed at 37 °C for 1 h to form the gel. Approximately 1 × 105 cells were mixed in an appropriate medium without FBS and dispensed into the upper chamber. Medium containing 10% FBS was added to each lower chamber. After incubation for 48–72 h, the number of invaded cells under the upper membrane was counted; cells remaining on the upper side of the upper membrane were removed, and the lower side of the upper membrane was stained with 20% methanol and 0.1% crystal violet.

### 2.8. Migration Assay

Cells were seeded into 6-well plates and scratched with a dedicated tip after 24 h. The medium was immediately changed after wounding, and a minimum of three parts were filmed for various periods (6–36 h). The migration distance was calculated using IMAGE J2 (https://imagej.net, accessed on 5 January 2020).

### 2.9. The mRNA Expression Analysis

mRNA expression of DAPK, Oct4, KLF4, Sox-2, and Nanog was analyzed by quantitative real-time PCR in KTC-1, MDAT-32, BCPAP, and 8505C cells. First, total RNA was isolated using the TRIzol^®^ RNA isolation reagent (Thermo Fisher Scientific, Waltham, MA, USA), and cDNA was then synthesized using a total of 1 ug of RNA. Next, real-time quantification of RNA expression was assessed using a 7500 Fast Real-Time PCR system (Applied Biosystems, Foster City, CA, USA). Each reaction mixture contained 1.0 μL of cDNA, 12.5 μL of QuantiFast SYBR Green PCR mixture, 1.0 μL of RNAse-free water, and 10 pM of each primer in a final volume of 25 μL. 18S ribosomal RNA was used as an internal control.

### 2.10. Immunoblot Analysis

Protein lysate was obtained from several cell lines and mouse, and human tissue samples using RIPA buffer. After measuring the concentration, 20–40 μg of total cell lysate was separated by protein size using a NuPAGE gel (Thermo Fisher Scientific, Waltham, MA, USA). The lysate was then transferred to a 0.45 μm nitrocellulose membrane (Amersham Bioscience, Piscataway, NJ, USA), which was blocked for 1 min using a dedicated Blocking Solution (Thermo Fisher Scientific, Waltham, MA, USA), reacted with the primary antibody, and incubated overnight at 4 °C. The membrane was washed with TBS-T (tris-buffered saline with Tween 20) and incubated with horseradish peroxidase-conjugated secondary antibody (1/5000) for 1 h at room temperature. After washing with TBS-T, enhanced chemiluminescence (Southern Biotech, Birmingham, AL, USA) was used to detect immune response proteins. The membrane fraction was performed with the 8505C cell using the Qproteome Cell Compartment Kit (Qiagen, 37502, Ann Arbor, MI, USA), and cytoplasmic and nuclear fraction samples were collected. DAPK1 (Invitrogen, Waltham, MA, USA, D2178), PathScanRMultiplex Western Cocktail I (Cell Signaling, Danvers, MA, USA, 5301), Oct4 (Santa Cruz Bio-technology, Dallas, TX, USA, sc-5279), KLF4 (Abcam, ab57030, Cambridge, UK, ab106629), Sox-2, Nanog (Cell Signaling Technology, Danvers, MA, USA, 2748, 4903), β-catenin (Millipore, Burlington, MA, USA, ABE208), active β-catenin (Millipore, Burlington, MA, USA, 05-665), Lamin A/C, GAPDH, and β-actin (Cell Signaling Danvers, Beverly, MA, USA, 2032, 2118, and 4970) antibodies were used.

### 2.11. Immunofluorescence

The MDA-T32 and 8505C cells were fixed with 4% paraformaldehyde, permeabilized, and blocked with 10% FBS for 1 h. The sample was then incubated with anti-Oct4 (dilution 1:200) or anti-β-catenin (dilution 1:200) antibodies overnight at 4 °C. Alexa 488 or Alexa 546 were selected as the secondary antibodies and counterstained with DAPI. Immunofluorescence images were taken using a confocal microscope (ZEISS, Oberkochen, Germany).

### 2.12. ALDEFLUOR Assay

The protocol was followed as described previously [23]. Briefly, cells were maintained for 10 days in an ultra-low adhesion 6-well plate, and then suspended in ALDEFLUOR assay buffer (STEMCELL Technologies, 01700, Cambridge, MA, USA) at a concentration of 10,000 cells/mL. Thereafter, 5 uL of DEAD (control) or activated ALDEFLUOR reagent was added to the sample tube, followed by incubation in a 37 °C incubator for 40 min. Cells were then pelleted by vortex at 3000 rpm for 5 min, re-suspended in 500 uL ALDEFLUOR buffer, and analyzed using a BD FACSA-II flow cytometer (Becton Dickenson, San Jose, CA, USA).

### 2.13. Immunohistochemical Staining

The degree of DAPK1 protein expression in PTC was evaluated by IHC with the anti-DAPK1 (D2178, Invitrogen, Waltham, MA, USA) antibody, performed on tissue microarray as previously described [23]. Cytoplasmic staining intensity was semi-quantitatively graded by an experienced pathologist (D.E.S.): 0, negative for expression; 1, weak expression; 2, moderate expression; 3, strong expression. The sample was classified as having positive DAPK1 protein expression if the intensity was graded above weak.

### 2.14. Thyroid Tissues

Twelve fresh-frozen PTC tissues from the Asan Bio Resource Center, Seoul, Korea were used for mRNA or protein expression analysis. This study protocol was approved by the institutional review board of Asan Medical Center.

### 2.15. Statistics

Statistics used in this experiment were performed using Student’s *t*-test for continuous variables and the Chi-square test for categorical variables. Continuous variables were expressed as mean ± standard deviation and categorical variables as numbers and percentages. A significant difference in variables among three or more groups was analyzed by one-way ANOVA. Statistical results and graphs were performed using GraphPad Prism version 5.1 (GraphPad Software INC., San Die, CA, USA). *p*-values of <0.05 were considered statistically significant.

## 3. Results

### 3.1. DAPK1 mRNA Expression in Thyroid Cancer

**Figure 1:**
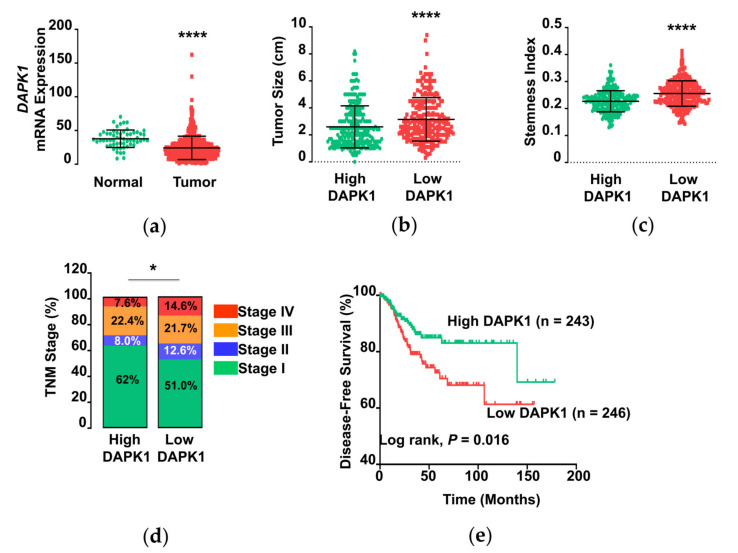
Low *DAPK1* expression is associated with poor clinical outcomes in human thyroid cancer. (**a**) Comparison of mRNA expression (TPM, transcripts per million) of *DAPK1* between normal thyroid (*n* = 59) and tumor tissue (*n* = 505). (**b**–**e**) Comparison of tumor size (**b**), stemness index (**c**), TNM stage (**d**), and disease-free survival (**e**) between high and low *DAPK1* tumor group. Transcriptome and clinicopathologic data from TCGA-THCA were analyzed. The disease-free survival was obtained using the Kaplan–Meier estimator. Data represent the mean ± SD. Asterisks (*p* < 0.05 (*) and *p* < 0.0001 (****)) indicate significant differences in the statistical analyses. Abbreviations: TCGA, the cancer genome atlas; THCA, thyroid carcinoma.

We first explored the clinical importance of the DAPK1 gene in human thyroid cancer by conducting a comprehensive analysis with a transcriptome and its matched clinicopathological data in the TCGA-THCA data. MRNA expression was significantly lower in tumors tissues compared with normal tissues (Figure 1a). When we divided the dataset into two groups according to DAPK1 expression levels, we observed a larger tumor size and higher stemness index (SI) in the low DAPK1 group (Figure 1b,c) than in the high DAPK1 group, indicating a higher proliferation rate and more stem cell-like features of these tumors, respectively. The low DAPK1 tumor group consistently displayed more aggressive features, including more advanced TNM (tumor-node-metastasis) stage, (Figure 1d) and lower DFS (Figure 1e). These findings suggest that DAPK1 is a tumor suppressor in thyroid cancer and its low expression is associated with tumor progression.

### 3.2. DAPK1 Inhibits Cell Invasion and Migration in Thyroid Cancer Cells

**Figure 2:**
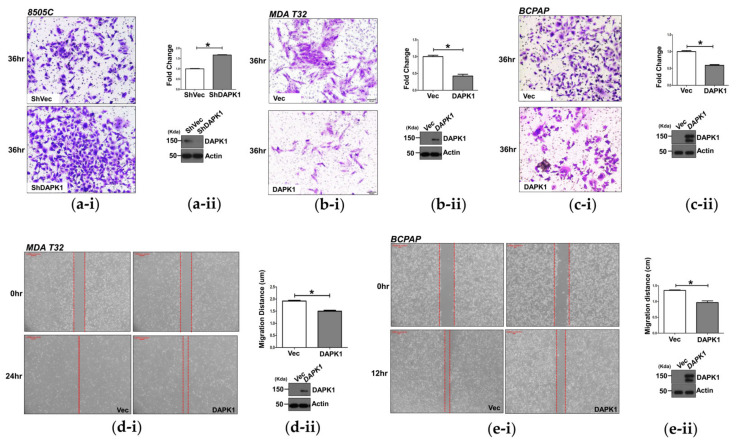
*DAPK1* is associated with the migration and invasion ability of 8505C, MDA-T32, and BCPAP cancer cells. (**a-i**) Transwell assays were performed in the 8505C cells after stable knockdown of *DAPK1*. A representative image was acquired 36 h after the initial seeding (pore size 0.8 μm). The image was acquired at a magnification of 1000×; scale bar represents 50 μm. (**a-ii**) Graph of the quantified results of (**a-i**). The sh*DAPK1*-8505C cells demonstrated significant migration compared with the shVector -8505C cells. The expression level of sh*DAPK1*-8505C cells was confirmed by Western blot. (**b-i**) Transwell assays were performed in the MDA-T32 after stable overexpression of *DAPK1*. A representative image was obtained from an image dyed 36 h after the initial seeding (pore size 0.4 μm). The image was acquired at a magnification of 100×; scale bar represents 50 μm. (**b-ii**) Graph of the quantified results of (**b-i**). The *DAPK1*-overexpressed MDA-T32 cells showed a significant decrease in invasion compared with the control. The expression level of *DAPK1*-overexpressed MDA-T32 cells was confirmed by Western blot. (**c-i**) Transwell assays were performed in the BCPAP after stable overexpression of *DAPK1*. A representative image was acquired 36 h after the initial seeding (pore size 0.4 μm). The image was acquired at a magnification of 100×; scale bar represents 50 μm. (**c-ii**) Graph of the quantified results of (**c-i**). The *DAPK1*-overexpressed BCPAP cells also showed a significant decrease in invasion compared with the control. The expression level of *DAPK1*-overexpressed BCPAP cells was confirmed by Western blot. (**d-i**) Wound-healing assays were performed in the MDA-T32 cells after stable overexpression of *DAPK1*. The representative image was acquired at the time of initial scratching and 24 h after. (**d-ii**) Quantification of the results of (**d-i**). The migration distance was calculated by comparing the gap distance of three different points at the initial and final time. The expression level of *DAPK1*-overexpressed MDA-T32 cells was confirmed by Western blot. (**e-i**) Wound-healing assays were performed in the BCPAP cells after stable overexpression of *DAPK1*. The representative image was acquired at the time of initial scratching and 12 h after the initial scratching. (**e-ii**) Quantification of the results of (**e-i**). The migration distance was calculated by comparing the gap distance of three different points at the initial and last time. The expression level of *DAPK1*-overexpressed BCPAP cells was confirmed by Western blot. Asterisks (*p* < 0.05 (*)) indicate significant differences in the statistical analyses. Each data point represents the mean ± standard error of three independent experiments.

We investigated the effects of DAPK1 in thyroid cancer according to TGCA data using 8505C, MDA-T32, BCPAP, and TPC-1 cells after confirming the basal protein level of DAPK1 (Supplementary Figure S1). We also analyzed the changes in cancer cell proliferation, migration, and invasion after modulating DAPK1 expression. DAPK1 protein expression was successfully downregulated in 8505C and KTC-1 cells (Figure 2, Supplementary Figure S2); its stable overexpression was confirmed in MDA-T32, BCPAP, and TPC-1 cell lines through Western blotting (Figure 2).

Cell proliferation decreased significantly, and colony formation after DAPK1 overexpression was induced by constitutively activating mutated DAPK1 transfection (ΔCaM) in 8505C cells (Figure 2a). Compared with the control, cancer cell invasion was significantly associated with DAPK1 expression in various thyroid cancer cells in siDAPK1 of TPC-1 cells (*p* < 0.001, Supplementary Figure S3a). Conversely, cancer cell invasion was significantly lower in DAPK1 ΔCaM-overexpressing MDA-T32 and BCPAP cells (*p* < 0.01 and *p* < 0.01, respectively, Supplementary Figure S3b,c). Consistent results were observed after KD of DAPK1 in thyroid cancer cells. Cell invasion increased in shDAPK1-8505C cells compared with shVec-8505C cells (*p* < 0.05, Figure 2a). In contrast, we observed a significant decrease in cancer cell invasion in MDA-T32 and BCPAP cells after DAPK1 overexpression compared with controls (*p* < 0.001 and *p* < 0.001, respectively, Figure 2b,c).

We also evaluated cancer cell migration after modulation of DAPK1 in these cells, which decreased significantly in stable DAPK1-overexpressing MDA-T32 and BCPAP cells (*p* < 0.001 and *p* < 0.001, respectively, Figure 2d,e). Consistent results were observed in TPC-1 cells after transient KD of DAPK1 using siRNA (*p* < 0.01, Supplementary Figure S3d). These results indicate that DAPK1 is a negative regulator of cancer cell invasion and migration in thyroid cancer cells.

### 3.3. DAPK1 Modulates Stemness in Thyroid Cancer Cells

**Figure 3:**
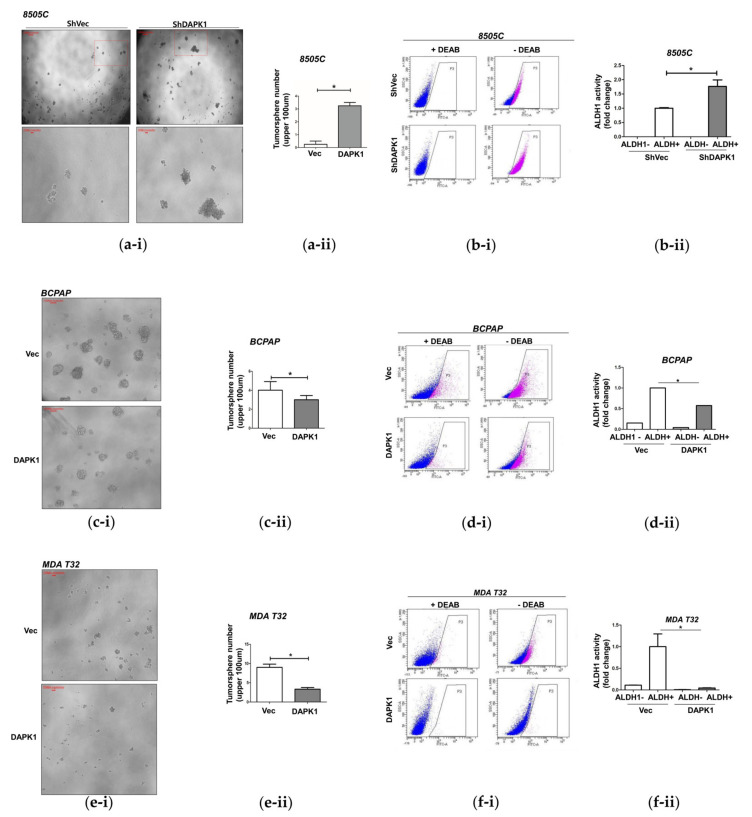
*DAPK1* is associated the migration and invasion ability of 8505C, MDA-T32, and BCPAP cancer cells. (**a-i**) *DAPK1* is associated with the stemness of thyroid cancer cells. (**a**,**b**) After 10 days of tumorsphere formation using shVector- and sh*DAPK1*- 8505C cells in low-attachment 6-well plates, the number of tumorspheres (**a**) and ALDH1 activity (**b**) were measured. (**a-i**) Compared with the control, the sh*DAPK1* 8505C cells showed a significant increase in tumorsphere formation ability. (**a-ii**) Quantification of (**a-i**). (**b-i**) ALDEFLUOR assay was used to determine ALDH1 activity. Compared with the control, ALDH1 expression increased significantly in the stable sh*DAPK1*- 8505C cells. (**b-ii**) Quantification of (**b-i**). (**c**,**d**) After 10 days of tumorsphere formation using GFP-Vector and GFP-*DAPK1* BCPAP cells in low-attachment 6-well plates, the number of tumorspheres (**c**) and ALDH1 activity (**d**) were measured. (**c-i**) Compared with the control, the stably overexpressing *DAPK1* BCPAP cells showed a significant increase in tumorsphere formation ability. (**c-ii**) Quantification of (**c-i**). (**d-i**) ALDEFLUOR assay was used to determine ALDH1 activity. Compared with the control, ALDH1 expression decreased significantly in the stably overexpressing *DAPK1* BCPAP cells. (**d-ii**) Quantification of (**d-i**). [24] After 10 days of tumor formation using GFP-Vector and GFP-*DAPK1* MDA-T32 cells, the number of tumorspheres (**e**) and ALDH1 activity (**f**) were measured. (**e-i**) Compared with the control, the stably overexpressing *DAPK1* MDA-T32 cells showed a significant increase in tumorsphere formation ability. (**e-ii**) Quantification of (**e-i**). (**f-i**) ALDEFLUOR assay was used to determine ALDH1 activity. Compared with the control, ALDH1 expression decreased significantly in *DAPK1*-overexpressing BCPAP cells. (**f-ii**) Quantification of (**f-i**). Asterisks (*p* < 0.05 [*]) indicate significant differences in the statistical analyses. Each data point represents the mean ± standard error of three independent experiments.

TCGA data indicated that low-level DAPK1 is closely related to higher SI; therefore, we next investigated the association between the DAPK1 level and stemness in thyroid cancer cells (Figure 3). We first analyzed tumorsphere formation given its close association with stemness. 8505C cells after stable KD of DAPK1 showed a marked increase in the number of tumorspheres (sized over 100 um) at day 10 compared with controls (*p* < 0.05, Figure 3a). Additionally, an ALDEFLUOR assay demonstrated greater stemness after DAPK1 KD in 8505C cells (*p* < 0.05, Figure 3b). Conversely, there was a significant decrease in the number of tumorspheres (>100 μm) at day 10 after overexpression of DAPK1 in BCPAP cells compared with controls (*p* < 0.05, Figure 3c). There was also a significant reduction in aldehyde dehydrogenase 1 (ALDH1) activity compared with controls (*p* < 0.05, Figure 3d). Consistent results were also observed in MDA-T32 cells. Tumorsphere formation was significantly reduced compared with controls in the presence of stable DAPK1 overexpression in MDA-T32 (*p* < 0.05, Figure 3e). Additionally, ALDH1 activity decreased significantly in cells overexpressing DAPK1 compared with control cells (*p* < 0.05, Figure 3f). The same trend was observed in TPC-1 cells. After transient KD of DAPK1, tumorsphere formation significantly increased in the siDAPK1 TPC-1 cells compared with controls (*p* < 0.05, Supplementary Figure S4a). These findings were also observed in shDAPK1 TPC-1 cells (*p* < 0.05, Supplementary Figure S4b). These data indicate that DKPK1 is a negative regulator of cancer cell stemness in thyroid cancer cells.

### 3.4. DAPK1 Regulates Cancer Stem Cell Properties by Modulating β-Catenin Activation

**Figure 4:**
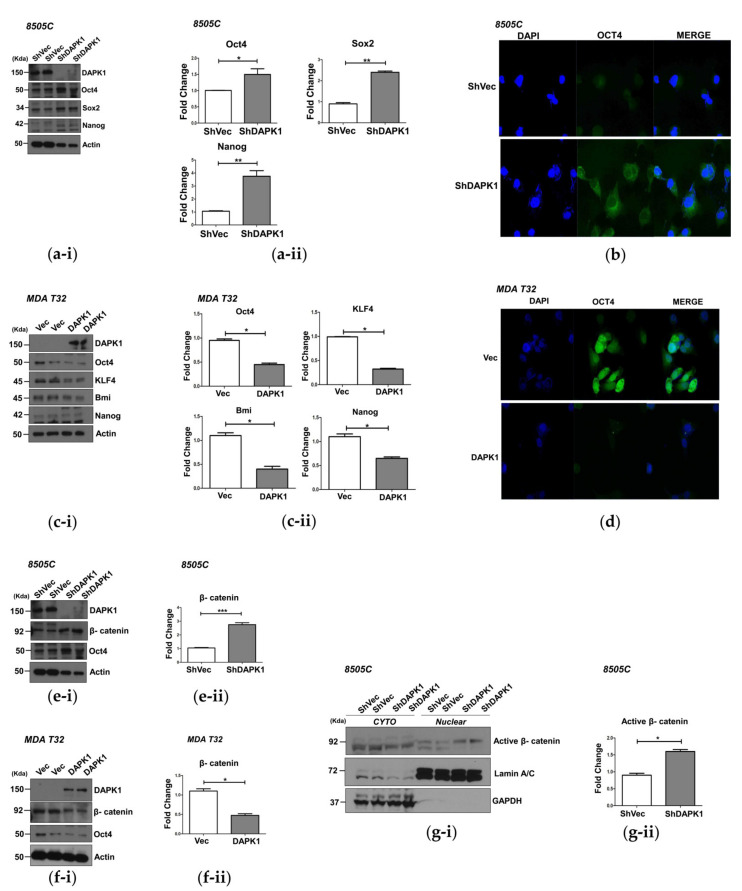
*DAPK1* is associated with the migration and invasion ability of 8505C, MDA-T32, and BCPAP cancer cells. (**a-i**) Transwell assays were performed in the 8505C cells after stable knockdown of *DAPK1*. A representative image was acquired 36 h after the initial seeding (pore size 0.8 μm). The image was acquired at a magnification of 100×; scale bar represents 50 μm. (**a-ii**) Graph of the quantified results of (**a-i**). There was significantly more migration of the sh*DAPK1*-8505C cells than the shVector -8505C cells. The expression level of sh*DAPK1*-8505C cells was confirmed by Western blot. (**b-i**) Transwell *DAPK1* is a negative regulator of thyroid cancer cell stem cell properties. (**a**,**b**) The expression of stem cell markers in 8505C cells after stable *DAPK1* knockdown. (**a-i**) Protein expression of *Oct4*, *Sox2*, and Nanog in *DAPK1*-stable KD 8505C cells. (**a-ii**) Quantification of the protein expression level. *Oct4, Sox2,* and Nanog expression increased significantly after *DAPK1* KD. (**b**) Immunofluorescence staining of *Oct4*, which showed increased cytoplasmic expression in shDAPK1-8505C cells compared with shVector-8505C cells. (**c**,**d**) The expression of stem cell markers in MDA-T32 cells after stable overexpression of *DAPK1*. (**c-i**) The protein expression of *Oct4, KLF4, Bmi,* and Nanog in *DAPK1*-overexpressing MDA-T32 cells. (**c-ii**) Quantification of the protein expression level, and the expression of *Oct4, KLF4, Bmi,* and Nanog decreased significantly after *DAPK1* overexpression in MDA-T32 cells. (**d**) Immunofluorescence staining of *Oct4*, which showed decreased cytoplasmic expression after *DAPK1* overexpression in MDA-T32 cells. (**e**–**g**) The β-catenin protein level in MDA-T32 and 8505C cells was investigated after *DAPK1* expression modulation. (**e-i**) The protein expression of β-catenin increased significantly in sh*DAPK1*- 8505C cells compared with the shVec-8505C cells. (**e-ii**) Quantification of the β-catenin protein level. (**f-i**) The protein expression of β-catenin significantly decreased in *DAPK1*-overexpressing MDA-T32 cells compared with the control. (**f-ii**) Quantification of the β-catenin protein level. (**g-i**) The protein expression of active β-catenin in the nucleus increased significantly in sh*DAPK1*-8505C cells compared with shVec-8505C cells. (**g-ii**) Quantification of the active β-catenin protein level in the nuclear fraction. GAPDH and lamin were used as loading controls for the membrane and nuclear fraction, respectively. Asterisks (*p* < 0.05 (*), *p* < 0.01 (**), *p* < 0.001 (***)) indicate significant differences in the statistical analyses. Each data point represents the mean ± standard error of three independent experiments.

We further investigated stem cell marker expression after modulation by conducting a Western blot, which confirmed significant increases in protein expression of the stem cell markers (Oct4, Sox2, and Nanog) after KD of DAPK1 in 8505 cells (Figure 4a). Immunofluorescence (IF) analysis revealed a significant increase in cytoplasmic Oct4 expression in DAPK1 KD 8505C cells compared with the control cells (Figure 4b). Significant decreases in protein levels of the stem cell markers (Oct4, KLF4, Bmi, and Nanog) were observed in the MDA-T32 cells stably overexpressing DAPK1 (*p* < 0.05, Figure 4c). A significant reduction of Oct4 expression in the cytoplasm was confirmed in DAPK1-overexpressing MDA-T32 cells compared with the controls (Figure 4d). Moreover, mRNA expression of stem cell markers decreased after DAPK1 overexpression in MDA-T32 and BCPAP cells but increased in DAPK1 KD in TPC-1 cells (Supplementary Figure S5a–c).

Beta-catenin regulates the expression of stem cell markers by binding to the promoter of these genes [25,26]. Therefore, we evaluated the change of β-catenin expression after modulation of DAPK1 expression. After stable KD of DAPK1 in 8505C cells, a significant increase in β-catenin protein expression occurred compared with the controls (Figure 4e); however, a decrease in β-catenin protein expression was observed in the DAPK1-overexpressing MDA-T32 cells (Figure 4f). We also confirmed that active β-catenin level in the nucleus increased significantly in *DAPK1* KD cells compared with control 8505c cells (Figure 4g). These data suggest that the DAPK1 regulates stem cell marker expression by modulating β-catenin activation.

### 3.5. The Association of DAPK1 Expression with Stemness Markers and Cancer Progression in Human Papillary Thyroid Cancer

**Figure 5:**
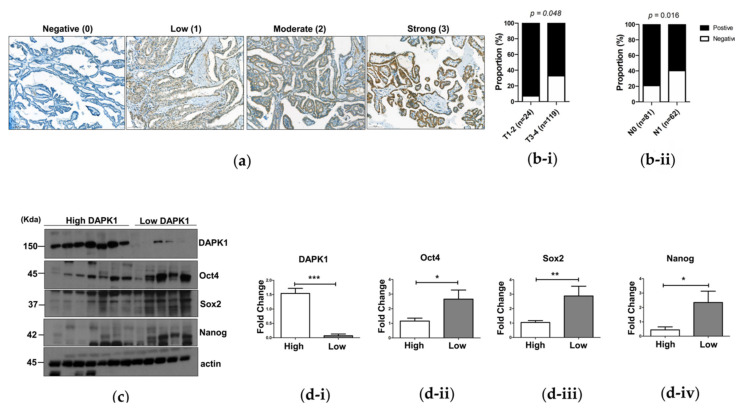
The relationship between DAPK1 and stem markers or pathological characteristics in thyroid cancer. (**a**) Representative images of IHC staining of *DAPK1* in PTC tissues. The cytoplasmic staining intensity was semi-quantitatively graded as negative (0), low (1), moderate (2), or strong (3). We regarded the *DAPK1* expression as positive when the tissues showed 1 or more staining intensities. (**b**) The relationship between *DAPK1* expression and pathological characteristics of PTC. (**b-i**) Comparison of the *DAPK1* expression between T1-T2 (*n* = 24) and T3-T4 (*n* = 119) tissues by the two-tailed Student’s *t*-test. The proportion of *DAPK1* positivity was significantly lower in T3-T4 tissues than T1-T2 tissues (*p* = 0.048). (**b-ii**) Comparison of the *DAPK1* expression between PTCs without LN metastasis (N0, *n* = 81) and with LN metastasis (N1, *n* = 62) by the two-tailed Student’s *t*-test. The proportion of *DAPK1* positivity was significantly lower in PTCs with LN metastasis than in those without LN metastasis (*p* = 0.016). (**c**,**d**) Western blot was performed to examine the expression of stem cell markers associated with the expression of *DAPK1* in human tissue samples. We used 12 fresh-frozen PTC human thyroid tissues. Actin was used as a loading control. (**c**) Representative Western blot results. The expression of stem cell markers (Oct4, Sox2, and Nanog) according to the expression of DAPK1 protein in human PTC tissues was investigated. When the protein expression of DAPK1 was high, the protein expression of various stem cell markers (Oct4, Sox2, and Nanog) was low in PTC tissues, and when the expression of DAPK1 was low, the expression of stem cell markers was confirmed to have increased conversely. (**d**) Quantification of (**c**). (**d-i**) DAPK1. (**d-ii**) Oct4. (**d-iii**) Sox2. (**d-iv**) Nanog Asterisks (*p* < 0.05 (*), *p* < 0.01 (**), *p* < 0.001 (***)) indicate significant differences in the statistical analyses.

We analyzed the association between DAPK1 protein expression and the clinicopathological characteristics of PTC with immunohistochemistry (IHC). A total of 143 patients with PTC were included in this study. Representative IHC images (for each intensity) are presented in Figure 5a. Among the 143 PTCs, 101 PTCs (71%) were positive for DAPK1 protein expression, which was higher in low-TNM-stage PTCs. T1–2 PTCs showed 93% positivity for DAPK1 protein expression, which was significantly higher than T3–4 PTCs (*p* = 0.048, Figure 5b). Additionally, PTCs with LN metastasis exhibited significantly lower DAPK1 expression than PTCs without LN metastasis (*p* = 0.016, Figure 5b). These findings are consistent with our in vitro and in vivo results and confirm that DAPK1 acts as a tumor suppressor in thyroid cancer, and may be a prognostic indicator of aggressiveness in PTC.

Finally, we performed a Western blot analysis to investigate the expression of stem cell markers related to DAPK1 expression in 12 fresh-frozen human PTC tissue samples. Representative Western blot results are presented in Figure 5c. We assessed the expression levels of Oct4, Sox2, and Nanog according to DAPK1 expression levels, which were roughly divided into high (*n* = 7) and low (*n* = 5) expressions. We observed increased expression of stem cell markers with lower expression of DAPK1. Quantification also indicated significance (*p* < 0.05, *p* < 0.01, and *p* < 0.05, respectively; Figure 5d). These findings confirmed the role of DAPK1 as a negative regulator of cancer stemness in thyroid cancer.

## 4. Discussion

In this study, we evaluated the role of DAPK1 as a regulator of cancer progression and stemness in thyroid cancer in vitro. Furthermore, we analyzed the association between DAPK1 expression and prognostic factors of PTC using TCGA data and immunostaining results in 143 PTC tissues. DAPK1 expression was significantly associated with lower LN metastasis, suggesting its role as a potential tumor suppressor in thyroid cancer progression. Secondly, DAPK1 may be a negative regulator of CSC-like properties in thyroid cancer. The negative association of DAPK1 mRNA expression with SI and DAPK1 protein levels with stem cell marker expression is indicative of the role of DAPK1 in cancer stemness. In the early stage of carcinogenesis, DAPK1 could inhibit cancer cell proliferation by regulating sensitivity to an apoptosis-related signaling pathway [27,28]. DAPK1 is primarily involved in metastasis rather than proliferation in the later stages of cancer progression by modulating cancer cell adhesion, migration, invasion, and immune evasion [12,18,28,29]. The role of DAPK1 in carcinogenesis could differ according to the type of cancer cell and the extent and the progression of cancer [12,18,28,29]. Our results indicate that DAPK1 is an important regulator of cancer stemness by controlling metastasis rather than cell proliferation in thyroid carcinogenesis.

CSCs, similar to somatic stem cells, have regenerative and differentiation capabilities and are thought to influence the recurrence of invasive growth and metastasis and impact drug resistance in the cancer process [17]. CSCs have been identified in many cancers, including breast, colon, gastric, and thyroid cancer, but their mechanism is not yet clearly understood [4,5,23]. Our study demonstrates that DAPK1 is closely associated with Oct4 regulation, which determines cancer stemness. Oct4 is a transcription factor that promotes other stem cell factors in the final stages of CSC development and is upregulated in breast, colon, and bladder cancers [17,30]. Oct4 has also been studied as a key component of drug-resistance mechanisms and cancer prognosis through its regulation of CSCs [17]. The expression of CSC markers, including Oct4, was negatively regulated by modulation of *DAPK1* expression in our study. Thus, tumorsphere formation and ALDH1 activity were also inhibited by DAPK1 in thyroid cancer cells. Our findings suggest that DAPK1 is a negative regulator of cancer stemness in thyroid cancer progression by regulating Oct4 expression.

Thyroid cancer is one of the most prevalent cancers worldwide [31,32]. Most thyroid cancers are well-differentiated and successfully treated with surgery, radioactive iodine, and thyroid hormone therapy. However, 5–10% of patients develop distant metastasis, and two-thirds of them become refractory with a poor prognosis [33,34]. CSCs have recently been identified as a crucial feature in some thyroid cancers underlying their aggression, recurrence, metastasis, and treatment resistance [33,35]. Previous studies have separated CSCs from thyroid cancer using Oct4 as a stem cell marker [35,36]. A recent study using Oct4 and Nanog to identify CSCs from anaplastic thyroid cancer cells revealed that these CSCs generate tumors in immunodeficient mice [37]. Another study identified a positive association between Oct4 expression or ALDH1 activity and tumor formation in vivo [36,38]. A significant increase in Oct4 and *ABCG2*, a drug-resistant gene, is also associated with EMT induction in thyroid cancer [39]. Therefore, CSCs would be a critical target for the treatment of refractory progressive thyroid cancer [33,35,40]. This study suggested that modulating DAPK1 could be a new strategy for targeting the CSC pathway in thyroid cancer.

Through the mechanism studied, DAPK1 promoted CSCs by regulating Oct4 level through the β-catenin pathway. Our findings align with earlier studies indicating that β-catenin regulates Oct4 expression [25]; the Notch pathway, Wnt/β-catenin, and hedgehog signaling are important in CSC expression [25,41,42]. β-catenin levels increased with downregulation of *DAPK1* and decreased with DAPKI overexpression in thyroid cancer cells, confirmed by confocal imaging [25]. Furthermore, active β-catenin significantly increased in the nucleus after KD of *DAPK1*. These results suggest that the DAPK1-β-catenin-Oct4 axis may be important for the stemness of thyroid cancer and that DAPK1 in the cytoplasm plays a role as a link between β-catenin and Oct4. However, further studies are needed to determine these regulatory mechanisms.

The other limitation of our study is the lack of validation of our findings via in vivo experiments. There are currently few studies that have elucidated the role of DAPK1 in carcinogenesis in vivo [12,16]. One study showed loss of DAPK1 in colorectal cancer cells enhanced tumor budding in vivo in the chorioallantoic membrane (CAM) model [12]. In the other study, DAPK1 overexpression inhibited tumor growth of prostate cancer cells in an in vivo xenograft model [16]. The mechanism differs in the two models in that the mechanism of CSC regulation is achieved by regulating Ca+ channels or by activating ZEB1, an important factor regulating EMT [12,16]. Future research is needed to elucidate the role of DAPK1 on thyroid carcinogenesis in vivo. However, the present study is beneficial because, for the first time, we evaluated the role of DAPK1 in regulating stemness in thyroid cancer with the validation of public data analysis.

## 5. Conclusions

In conclusion, DAPK1 plays an important role as a tumor suppressor in thyroid cancer migration, invasion, and metastasis. Additionally, DAPK1 significantly modulates the reprogramming of stem cell markers by regulating β-catenin activation. DAPK1 may therefore be an independent prognostic marker and therapeutic target in thyroid cancer.

## Data Availability

The authors confirm that the data supporting the findings of this study are available within the article and its Supplementary Materials. Additional raw data supporting the findings of this study are available from the corresponding authors (M.J.J. and W.G.K.) on request.

## Supplementary Materials

The following are available online at https://www.mdpi.com/article/10.3390/cells10112994/s1, Figure S1: The protein level of *DAPK1* in thyroid cell lines; Figure S2: The effect of *DAPK1* on the proliferation of thyroid cancer cells; Figure S3: The effect of *DAPK1* on the migration and invasion ability of TPC-1 and MDA T32 cell lines; Figure S4: The effect of *DAPK1* on the tumorsphere formation; Figure S5: The effect of DAPK1 on the stemness at mRNA changes.
