# Supplementary material for: Death-Associated Protein Kinase 1 Inhibits Progression of Thyroid Cancer by Regulating Stem Cell Markers
Source: Cells. 2021 Nov 3;10(11):2994. doi: 10.3390/cells10112994 (PMC8616132; doi:10.3390/cells10112994)

Supplemental data

Article

Death-Associated Protein Kinase 1 Inhibits Progression of Thyroid Cancer by Regulating Stem Cell Markers

Mi-Hyeon You [1], Woo Kyung Lee [2], Meihua Jin [1], Dong Eun Song [3], Sheue-yann Cheng [2], Tae Yong Kim [1], Won Bae Kim [1], Min Ji Jeon [1,*] and Won Gu Kim [1,*]

[1] Department of Internal Medicine, Asan Medical Center, University of Ulsan College of Medicine, Seoul 05505, Korea; mhyou80@gmail.com (M.H.Y.); meihua711@naver.com (M.J.); tykim@amc.seoul.kr (T.Y.K.); kimwb@amc.seoul.kr (W.B.K.)

[2] Laboratory of Molecular Biology, Center for Cancer Research, National Cancer Institute, National Institutes of Health, Bethesda, MD 20892, USA; wookyung.lee@nih.gov (W.K.L.); Chengs@nih.gov (S.Y.C.)

[3] Department of Pathology, Asan Medical Center, University of Ulsan College of Medicine, Seoul 05505, Korea; hipuha@hanmail.net (D.E.S.)

* Correspondence: mj080332@gmail.com (M.J.J.); wongukim@amc.seoul.kr (W.G.K.); Tel.: +82-2-3010-1317 (M.J.J.); +82-2-3010-5883 (W.G.K.); Fax: +82-2-3010-1317 (M.J.J.); +82-2-3010-6962 (W.G.K.)

Supplemental Figure S1. The protein level of *DAPK1* in thyroid cell lines. Tubulins were used as a loading control.

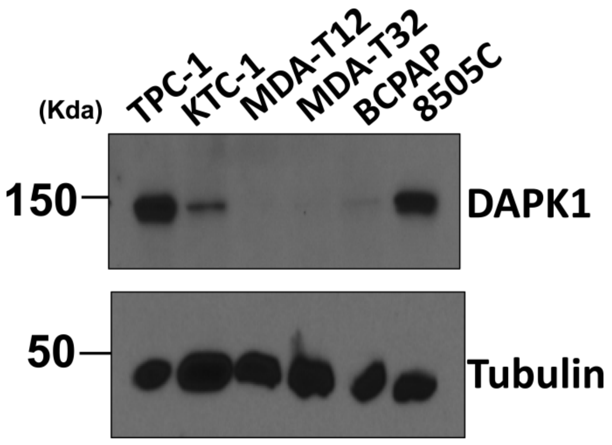

Supplemental Figure S2. The effect of *DAPK1* on the proliferation of thyroid cancer cells.

(a-i) Proliferation analysis of 8505C cells after transient transfection of ΔCaM.

(a-ii) Colony formation of 8505C cells on day 7 after transient transfection of ΔCaM.

Asterisks ($p < 0.05$ [*], $p < 0.01$ [**], $p < 0.001$ [***]) indicate significant differences in the statistical analyses. Each data point represents the mean ± standard error of three independent experiments.

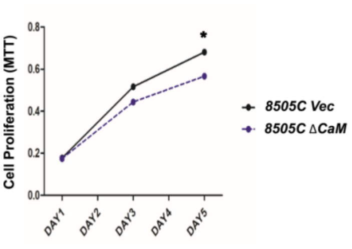

(a-i)

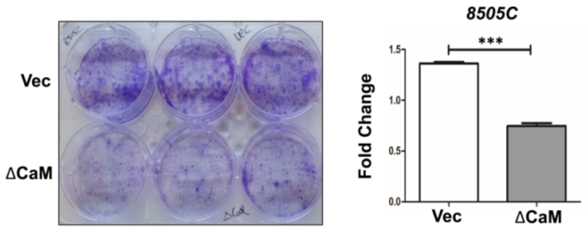

(a-ii)

Supplemental Figure S3. The effect of *DAPK1* on the migration and invasion ability of TPC-1 and MDA T32 cell lines

(a-i) Transwell assays were performed in the TPC-1 cells after transient knock-down of *DAPK1*. A representative image was obtained from an image dyed 36 h after the initial seeding (pore size 0.8 μm). The image was acquired at a magnification of 100x; scale bar represents 50 μm.

(a-ii) Graph of the quantified results of (a-i). The si*DAPK1*-TPC-1 cells significantly migrated more than the siVector –TPC-1 cells.

(b-i) Transwell assays were performed in the MDA-T32 cells after transient ΔCaM transfection. A representative image was acquired 24 h after the initial seeding (pore size 0.8 μm). The image was acquired at a magnification of 100x; scale bar represents 50 μm.

(b-ii) Graph of the quantified results of (b-i). The ΔCaM transfected MDA-T32 cells also showed significant decrease in invasion compared to the control.

(c-i) Transwell assays were performed in the BCPAP cells after transient ΔCaM transfection. A representative image was acquired 24 h after the initial seeding (pore size 0.8 μm). The image was acquired at a magnification of 100x; scale bar represents 50 μm.

(c-ii) Graph of the quantified results of (c-i). The ΔCaM transfected BCPAP cells also showed significant decrease in invasion compared to the control.

(d-i) Wound-healing assays were performed in the TPC-1 cells transient knock-down of *DAPK1*. The representative image was acquired at the time of initial scratching and 20 h after the initial scratching.

(d-ii) Quantification of the results of (d-i). The migration distance was calculated by comparing the gap distance of three different points at the initial and last time.

Asterisks ($p < 0.05$ [*], $p < 0.01$ [**], $p < 0.001$ [***]) indicate significant differences in the statistical analyses. Each data point represents the mean ± standard error of three independent experiments.

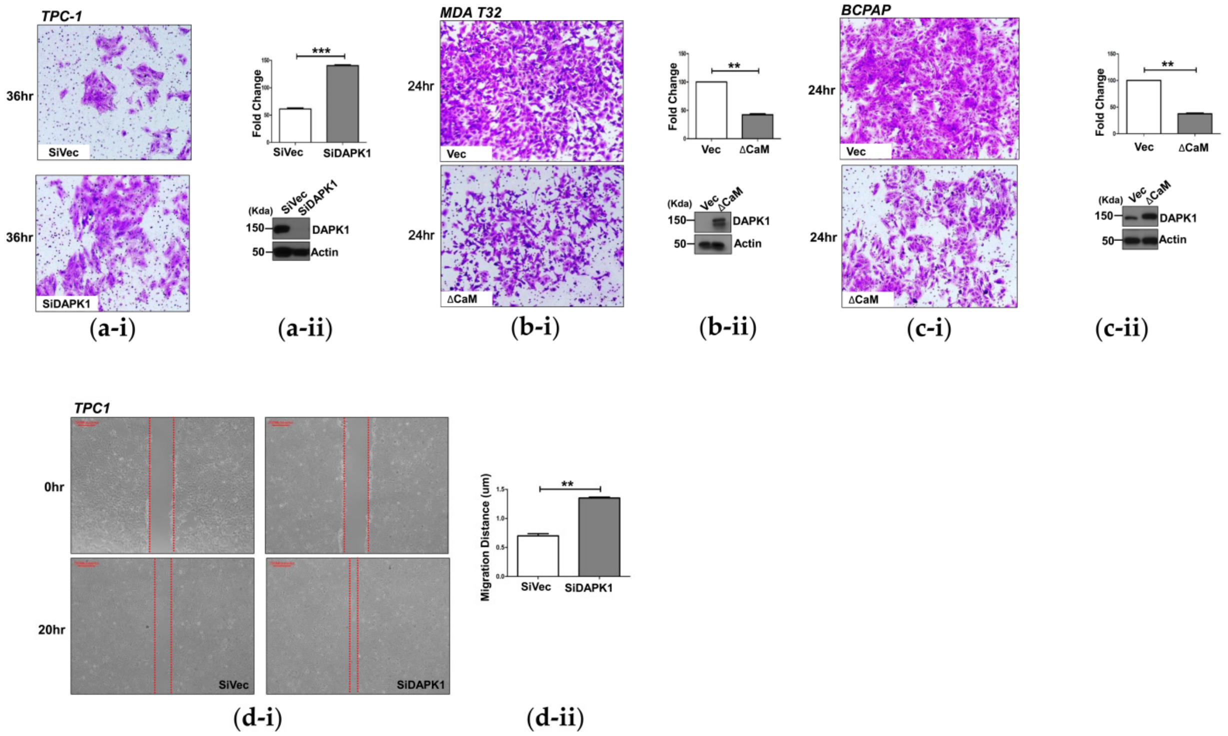
TPC-1
36hr
SiVec
36hr
SiDAPK1
Fold Change
***
SiVec
SiDAPK1
(Kda)
150
50
DAPK1
Actin
MDA T32
24hr
Vec
24hr
ΔCaM
Fold Change
**
Vec
ΔCaM
DAPK1
Actin
BCPAP
24hr
Vec
24hr
ΔCaM
Fold Change
**
Vec
ΔCaM
DAPK1
Actin
(a-i)
(a-ii)
(b-i)
(b-ii)
(c-i)
(c-ii)
TPC1
0hr
20hr
SiVec
SiDAPK1
Migration Distance (um)
**
SiVec
SiDAPK1
(d-i)
(d-ii)

Supplemental Figure S4. The effect of *DAPK1* on the tumorsphere formation

(a-b) After 10 days of tumorsphere formation using DAPK1 knock-down (KD) TPC-1 cells in low-attach 6 well plate, the number of tumorspheres were measured. (a-i) Transient KD of *DAPK1* in TPC1 cells showed a significant increase in tumorsphere formation ability. (a-ii) is the quantification of (a-i). (b-i) Stable KD of *DAPK1* in TPC-1 cells also showed a significant increase in tumorshpere formation ability. (b-ii) is the quantification of (b-i).

Asterisks ($p < 0.05$ [*], $p < 0.01$ [**], $p < 0.001$ [***]) indicate significant differences in the statistical analyses. Each data point represents the mean ± standard error of three independent experiments.

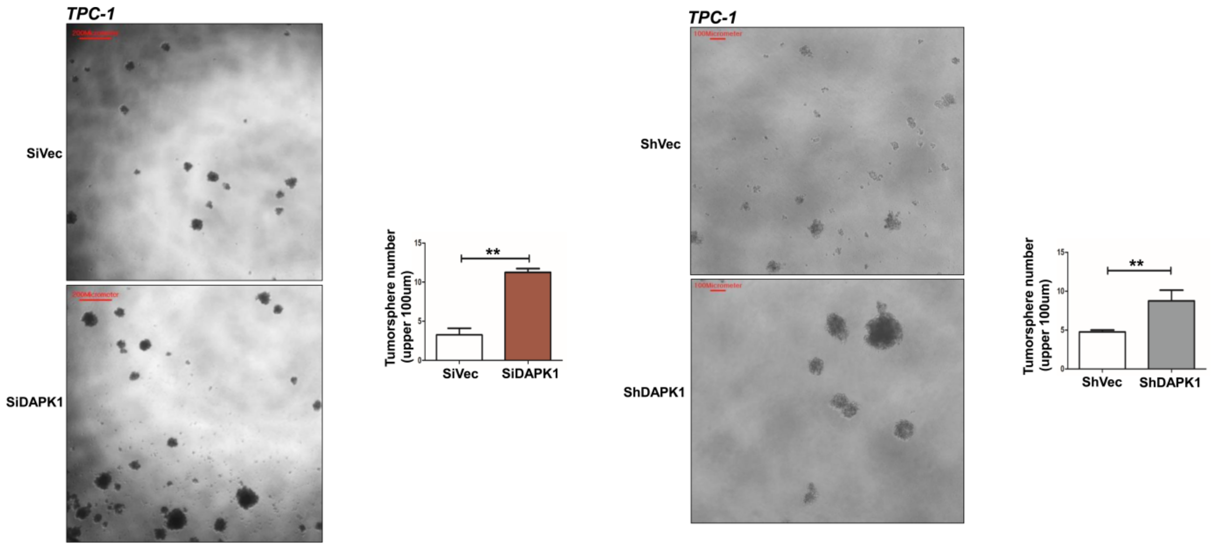

(a-i) **(a-ii)** **(b-i)** **(b-ii)**

Supplemental Figure S5. The effect of *DAPK1* on the stemness at mRNA changes

(a) mRNA expression of *Oct4* (a-i), *KLF4* (a-ii), *Sox2* (a-iii), and *Nanog* (a-iv) in MDA-T32 cells after *DAPK1* stable over expression. (b) mRNA expression of *Oct4* (b-i), and *KLF4* (b-ii) in BCPAP cells after *DAPK1* stable over expression. (c) mRNA expression of *Oct4* (c-i), *KLF4* (c-ii), *Sox2* (c-iii), and *Nanog* (c-iv) in TPC-1 cells after stable knock-down of DAPK1. 18S was used as an internal control.

Asterisks ($p < 0.05$ [*], $p < 0.01$ [**], $p < 0.001$ [***]) indicate significant differences in the statistical analyses. Each data point represents the mean ± standard error of three independent experiments.

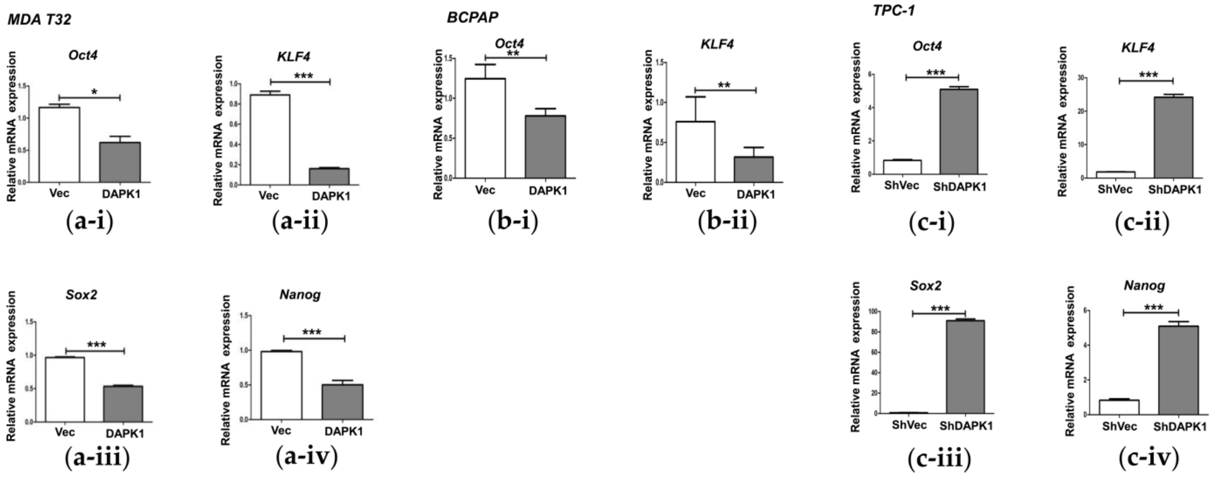

Supplement: Supplementary file 1 [file cells-10-02994-s001.zip › cells-1439841-supplementary.pdf]
